# Fruits and Farinacea, the Proper Food of Man

**Published:** 1846-04

**Authors:** 


### Fruits and Farinacea, the proper Food of Man. By *John Smith.* Octavo, pp. 422. London, 1845.

It is quite refreshing, amid our ordinary critical labours, to meet with the work of a honest enthusiast. Such is Mr. Smith, of Malton; and although we cannot agree with the conclusions he arrives at, and should feel very averse to following the practice he inculcates, we must admit he has argued his case well and ably, and has produced by far the best work upon an interesting subject. His object is to recommend an exclusively vegetable diet as the original, natural, and best diet of man, and he ransacks the fields of comparative anatomy, chemistry, personal testimony, and national peculiarities, to furnish due and fitting arguments and evidence. He is moreover no mere theorist, but practises what he preaches, and we cannot do better than commence this notice with a brief account of his experience. Having been engaged in the preparation of an Essay which brought prominently before his notice the similarity in the development of the nervous system in man and the higher classes of animals, he was induced to ask himself the following question:

"Is man justified in slaughtering animals for his food; seeing that, by means of a beautifully organized structure, they are rendered exquisitely sensible both of pleasure and pain? The answer I mentally returned to the inquiry was—If the flesh of animals be necessary to the health, happiness, and longevity of man, then the law of self-preservation will warrant his taking the life of animals; provided he be guilty of no cruelty, nor cause unnecessary pain to the animal that he sacrifices to supply his wants; but if, upon further inquiry, it should appear, that the life of man can be preserved, his happiness or pleasure continued or rendered more pure and satisfactory, and the period of his mortal existence unabbreviated or prolonged, by a diet of which the flesh of animals forms no part—then would neither wisdom nor benevolence sanction the horrid cruelties that are daily perpetrated, in order to pamper the perverted appetites of man."

To determine this question he entered upon an elaborate train of investigation—the steps and results of which are detailed in the present work.

"I arrived at the firm conviction, that the flesh of animals is not only wholly unnecessary, but decidedly prejudicial to man's health and well-being. I therefore at once discontinued it, as an article of diet; and, notwithstanding the expressed fears and remonstrances of my friends, I persevered; and was soon rewarded with better health and more real enjoyment, than I had experienced during many previous years. Having derived incalculable advantages from a strict adherence to a fruit and farinaceous diet, and being fully satisfied (after a long and patient investigation of evidence) that it is a food adapted to *all* constitutions, in *all* climates fit for the residence of man, I can no longer resist the importunity of my friends to make known to the public the result of my inquiries. * * * * * * * * About nine years ago I suffered very much from dyspepsia; and was treated *secundem artem*, by my medical adviser, who was eminent in his profession; but I derived little benefit from either the diet or medicine prescribed for me. I adopted a vegetable diet, not as a remedy for my complaint, but for the reasons already mentioned; and after using this regimen for a very short period, I no longer suffered from a disease that had formerly been a daily and severe drawback upon the pleasures of existence. Like the patients mentioned by Mr. Thackrah, I have often resumed my flesh-eating habits; partly for the sake of experiment, and partly with a view of complying with the general usages of society, and to avoid singularity; but, after a short time, I have always had cause to repent the change, from the inconvenience and pain that were the consequence. I have now sufficiently tested the diet practically; and hesitate not to say, that since I have totally abstained from animal food, I have possessed more health and strength of body, more peace and serenity of mind, as well as more intellectual enjoyment, than at any former period of life: and I trust that I shall never once be induced to depart from that simple mode of living, which, while it has conferred on me the inappreciable advantages just mentioned, also yields more exquisite sensual gratification than I ever experienced on the most richly-flavoured dishes of a former period."

When we say the author manages his question ably, we would be understood that he does so in the sense rather of an acute advocate than an impartial judge; for, while he submits all evidence on the other side to rigid scrutiny, he receives any testimony in favour of his own view from any quarter, trustworthy or not (and such as sometimes amounts to mere coincidence), as a matter of course. He divides his work into three parts, each leading naturally to the other; namely, into an inquiry as to what constituted the *original* food of man; what is his *natural* food; and what is his *best* food: winding up with a few reasons for believing that a vegetable millenium is not to be despaired of, although not immediately to be anticipated.

1. The section upon the *original food* we may at once dismiss as of no more consequence to us at the present day than the original clothing or original language of man. Moses and the classic poets, according to Mr. Smith, show plainly enough that man was originally a vegetable feeder; and if they had not done so, he says we might have inferred as much from a contemplation of his innocent condition and the original purity of his sensual perceptions. Had not the book contained better matter than such stuff as this we should not have troubled our readers with a notice of it.

2. What is the *natural food* of man? To answer this, Mr. Smith glances hastily at the structure of the alimentary organs of the carnivora and herbivora as compared with those of man, and arrives at the conclusion that man, in this respect, most closely resembles his brother the ape, and as this is for the most part a frugivorous animal, so naturally is he. Were man destined to live in the wild state like other animals, food of some such description is that which he would most readily obtain in consequence of the feebleness of his jaws, the absence of powerful tearing or grinding teeth, and his destitution of natural weapons of offence and defence, and such food would comport well enough with the structure of his alimentary canal. But the possession of reason has enabled him to conquer articles of diet from every domain, and the art of cookery, also peculiar to him, renders those of them which in their crude state would be ill adapted to nourish him competent to effect this object. But Mr. Smith observes the facts, of so large a variety of substances agreeing thus with man, and his being able to thrive well upon all or any of them under different circumstances, prove not his *omnivorous* nature, or that they are naturally adapted to his sustenance. What do they prove then ? only his great *adaptability*. Be it so ; and surely an acknowledged adaptability to his ever-changing circumstances, so important a feature in the natural history of man, is a sufficient admission, which renders idle any discussion as to what might have been his especial adaptation under circumstances in which he has never been placed. The statement that his digestive organs are essentially adapted for vegetable diet is a mere assumption.

After maintaining that animal food must *naturally* be very repugnant to the senses of sight, smell, and taste, and explaining the fact of its being *actually* the very contrary of this by the force of habit which has blunted natural sensibilities, and engendered most artificial ones—the author takes leave of this part of his subject by a disquisition upon its moral aspect, endeavouring to prove that the slaughtering animals for the purposes of food must do violence to the sensitive and moral feelings of man. This argument against the use of animal food on account of the cruelty of the practice is evidently a weak one, and one we never expected to have seen revived after the slaughter of Ritson's work upon the subject by the Edinburgh Reviewers. It may perhaps with some persons answer the author's purpose to parade tales of the tormenting animals prior to butchering them, cutting slices out of the buttocks of the live Abyssinian oxen, or the slow roasting of the Strasbourg geese; but while every one will join him in deprecating the cruelties here stigmatized, or any others engendered by epicurism, all reasonable people are aware that, as a general rule, animals are killed for food with the infliction of as little suffering as possible; while the statement of the fact that, numberless creatures are reared solely for the purpose of supplying man with food, and pass their lives in well-cared-for ease and comfort, is but the expression of the existence of a vast amount of animal happiness, which but for this circumstance would never have been created, and which is chequered and even terminated with less physical suffering than man himself has to endure.

3. The former portions of the subject are but preparatory and subordinate to the question, *Which is the best food of man ?* the only one indeed of any practical interest whatever. Mr. Smith distributes his proofs, that a vegetable diet is the most appropriate one, over several chapters, some of which we may notice.

*Vegetables contain all the elements and qualities necessary for the complete nutrition of man.*—This requires no elaborate proof, seeing that whole nations subsist upon vegetable matters alone. Nevertheless, the researches of modern chemists have proved the complete identity of the protein derived from animal and vegetable sources. Mr. Smith, while admitting in general the correctness of Liebig's views on the nature of food and the nutrition of animals, demurs to that portion of them which denies to the non-azotized principles the power of supporting life by nourishing the tissues, and assigns to them only the province of maintaining the animal heat. If this were the case, the diet of the inhabitants of tropical regions, abounding as it does in carbon and hydrogen, would generate a quantity of caloric far beyond their means of dissipation. He maintains that nitrogen is introduced into the system by other means than as a component of articles of food, and especially during insalivation and deglutition. This, in the stomach and duodenum, plays an important part in the combinations it forms, according to the exigencies of the system, with the otherwise superabundant proportions of carbon and hydrogen. As the author makes great use of these very questionable views in various parts of his work, we will extract some of his observations upon them.

"Neither men nor the lower animals are at all times so situated as to be able to procure, in sufficient abundance, that food which contains all the elements in the precise proportion and mode of combination best suited to their organization; the atmosphere, therefore, presents an immense reservoir, always at hand to make up deficiencies by means of mastication or respiration; and the digestive, chylopoietic, and secerning organs are endowed with such capabilities as to vary, within certain bounds, their proper functions; and to seize, with unerring precision, those elements of the atmospheric air of which the ingesta and circulating fluids are deficient.

"In applying these views to man, on a natural or vegetable diet, the following suggestions are offered. In warm climates, where an elevated temperature is incompatible with great muscular exertion, nature has provided a bountiful and pleasant repast of fruit, rice, and other vegetables possessing a considerable proportion of carbon and hydrogen, but little nitrogen. By virtue of affinities modified by vital agency, these nutritive substances are formed (in the stomach, duodenum, &c.) into new compounds by a re-arrangement of their elements, and by a combination with those of the atmosphere; thus producing either proteine or fat, as the wants of the system may determine. If the tissues are wasted by exercise, more oxygen and nitrogen are supplied by the atmosphere, so as to prevent the formation of oleaginous compounds; and the albuminous principles that result, are converted into fibrin to renovate the system; but, if sedentary occupations preponderate, less fibrin becomes necessary; the deficient supply of air causes more oxygen to be separated from the food; and an increase of fat is the consequence, especially if the food be in excess. If a more azotized diet be indulged in, then—as there is less occasion for the formation of the protein from the starch—the carbonaceous compounds must be eliminated by the skin, liver, and lungs; but, as the cutaneous surface, especially of the white variety of mankind, is not constituted for performing the additional duty now demanded of it, and as in these circumstances there is a deficient supply of oxygen to the lungs, carbon accumulates in the blood; and the liver is called into an excessive exercise of its function, in consequence of the inactivity of the skin and lungs. Hence the prevalence of hepatic disease in hot climates.

"In cold and temperate regions, wheat and other azotized products may be more freely indulged in; and the carbonaceous principles of food are then left at liberty for the respiratory function; muscular exercise becomes more easy and pleasant, and caloric is more abundantly formed. The inhabitants of these countries are more exposed to diseases of the chest, and their numerous train of distressing complaints arising from the presence of an abnormal proportion of lithic acid in the system, such as gout, rheumatism, gravel, &c. The extreme indulgence in animal food in these countries becomes the predisposing cause of all these diseases, as well as of dyspepsia and liver-complaints. If flesh, or other highly azotized food, be taken with a very small proportion of starchy matter, the sufferings of the dyspeptic are alleviated, because there is then less carbon for the liver to separate; but this diet demands more exercise from the lungs, in consequence of the diminished supply of oxygen from the food, and hence its danger to persons threatened with phthisis, as well as to gouty individuals, from its favouring the production of lithic acid. If the dyspeptic were to entirely abandon the use of animal food, and adopt a diet of fruit and farinacea, not only would the disease be palliated (as in the above treatment), but in the generality of cases entirely cured, without throwing an additional burden upon the lungs or kidneys; the former having their labour remitted by the disengagement of oxygen from the food, during the conversion of starch into protein; and the latter having less duty to perform, in consequence of the diminished supply of substances containing protein ready formed." 160.

Mr. Smith endeavours to meet the prevalent opinion of the indigestibility of *fruit*, by the considerations—1, that it is so opposite a diet to that which most persons have accustomed themselves, and hence, at first, disorders; 2, it is only eaten at dessert, when the stomach is already filled with various incongruous aliments; 3, it is eaten with imperfect mastication and insalivation.

There are many very excellent observations deprecatory of taking food in too concentrated a state, thus overlooking the important combinations of innutritious and nutritious matter with which Nature furnishes us. The ill-effects of this are undesignedly shewn in Stark and Majendie's experiments. We have only space for a few observations relating to *bread*:—

"Knight, in his Physiological and Horticultural Papers, says, 'Bread made of wheat, when taken in large quantities, has probably, more than any other article of food in use in this country, the effect of overloading the alimentary canal; and the general practice of French physicians points out the prevalence of diseases thence arising among their patients.' All the evils said to be produced by living on bread, are due to our modes of refining upon nature; and though it must be admitted, that bread made from the finest wheaten flour, if eaten in great abundance, and without a due admixture with innutritious matter, will be productive of serious consequences to health, yet it can be shown upon good authority, that many individuals have subsisted for years on coarse, undressed, wheat-meal bread and water alone; and have not only improved in health, but become remarkably robust and vigorous. Children whose food, for a considerable time, consists of superfine flour-bread, arrowroot, and other concentrated substances (such as sugar, butter, &c.), may appear fat and well; but do not acquire strength; they generally become weak and sickly, and are often covered with sores. Hence, some physicians, who have written on the diet of children, have spoken in severe terms against confining children to an exclusively vegetable diet. But if a child be put upon a diet of good bread, made of undressed wheat-meal, with milk-and-water, or pure soft-water for drink, and be allowed to indulge pretty freely in the use of good fruits in their seasons, none of the evils which result from concentrated forms of aliment, or which are attributed to vegetable diet, will be experienced; but the child, if in other respects properly treated, will be healthy, robust, and sprightly." P. 176.

Passing over the next two chapters, which are occupied in exhibiting the excellent effects a vegetable diet has exerted upon not only individuals but whole nations, we come to that which maintains that such a diet is

*consistent with physical strength and activity.* Mr. Smith seems to view the animal frame as the depositary of a certain amount of vital power, which will either be exhausted soon or late, according to the extravagance or care with which it is expended. Thus, under the use of animal food, the processes of assimilation and nutrition are more rapidly performed, and the waste of organised matters proportionally great, as is seen in the more speedy recurrence of the sensation of hunger, and the necessity of renewed supplies of food. By its stimulating effects it may enable great efforts to be made through a brief period; but these are followed by proportionate exhaustion; and if prolonged, sustained, truly effectual, exertion is required, then does vegetable food best enable it to be made; while, if we seek for a consentaneous development of the physical, mental, and moral powers, then is it essential. Numerous instances of great power of endurance, occurring among nations who are vegetable feeders, are adduced; it not being Mr. Smith's object, however, to deny that great bodily vigour and strength may be produced by a mixed diet, but to shew that the possession of these is not inconsistent with subsistence upon a vegetable one. The case of the English labourer or navigator, whose superior physical powers is so well appreciated by the contractor of foreign railways and canals, and which, (as also the energy and power of the English operative classes in general, whose like exists nowhere), has been usually, and we believe correctly, attributed in great part to his better and more animalized diet, is endeavoured to be gotten over by the author by the declaration, that however unable the liver upon grain, fruit, and roots is to cope with his carnivorous rival during the brief periods of great toil, yet that, owing to greater continuity of his lesser modicum of exertion, he is in the end able to accomplish more—a most gratuitous and unfounded assertion. Indeed, we are persuaded that just the contrary is the fact, and that a man laboriously employed and living upon a mixed diet, and avoiding excess of every kind, will be enabled to continue his exertions over a much longer period than one unprovided with such food. Of course we are here speaking of the inhabitants of temperate climes. It is true that the British and Irish peasantry are hard-working, laborious, races, and their food only vegetable; but who does not believe that their present great liability to disease and premature mortality would be much diminished could a better diet be provided for them?

*Animal Food a Cause of Disease.*—Mr. Smith makes a round assertion that animal food is a leading cause of our diseases, just as a vegetable regimen is one of the approved means of their removal. His remarks are only just when applied to some of the easier and indolent classes of society, who doubtless consume more animal food than their habits of exercise, &c. justify; but which of the diseases of the hard-working classes of the community can we justly refer to such a source? and what medical practitioner is unable to point out numerous cases evidently due to a privation of this, or at all events relieved by having recourse to it? But looking at the matter in a general point of view, we may ask Mr. Smith how comes it, if animal food is so detrimental to health, that our amount of mortality is continually on the decline, notwithstanding that the consumption of butcher's meat is, as regards the mass of the people, infinitely greater than when this was higher?

Gout, of course, is a strong card in our author's hand, when detailing the diseases arising from the use of animal food, but he makes no allusion to another frequent element in its production—the too free indulgence in vinous compotations. We entirely demur to his statement, that animal food is a common cause of scrofula and consumption. If this were the case, surely these diseases should not commit such cruel devastations among the vegetable-consuming portions of the Irish, Scotch, and French.

*Beneficial Effects of Vegetable Food on Invalids.*—The experiment of treating *Dyspepsia* by an exclusively vegetable diet seems to have been rather extensively undertaken in America; and, according to the report of Dr. North of Hertford, with great success, although he does not specify the class of cases benefited. The profession in this country usually condemning such regimen, few cases of its adoption are recorded. The author, however, quotes two or three from Dr. Abercrombie and Mr. Thackrah. His own case we have already adverted to; and the following is an abstract furnished by that veteran vegetable feeder, Dr. Lambe.

"From the age of 19 to 35 I was constantly suffering from the usual symptoms of dyspepsia; which, towards the latter period, were accompanied by a constant and oppressive pain about the stomach. At the age of 35 I had an attack of enteritis, which was severe enough to require two venesections; after this I never went out in the damp of the evening, without feeling some tenderness of the abdomen. Under these circumstances, together with a general feebleness of health, I determined to try the effect of substituting distilled water for common water as my drink. The effect of the change was a thorough relief of the dyspeptic pains and abdominal tenderness. In the ensuing three years, a headache, from which I had suffered occasionally earlier in life, returned so frequently and so severely, as to induce me to take active measures for its relief. I then determined to abstain from animal food, as well as from common water. The intensity of the paroxysms was instantly relieved; yet they recurred, in a mitigated form, for at least 30 years. I have been engaged in the active duties of my profession until the middle of last year, which was the 80th of my life. Since then, from a partial failure of my sight, I have retired into the country; where, making allowance for my time of life, I enjoy a good share of health." P. 281.

Mr. Smith enumerates the diseases (scrofula, scurvy, epilepsy, dysentery, inflammation, ulcers, &c.) relievable by vegetable diet in rather too indefinite and quack-doctor a style for our taste. An isolated case or two of this or that disease, successfully treated by this or any other means, to the medical observer says nothing whatever. That vegetable diet is a valuable adjunct in the treatment of many or most diseases, none will deny; but to rely upon it exclusively, or to adopt it in all cases, would augur but little wisdom on the part of the practitioner.

Mr. Smith maintains that a vegetable regimen exerts an especial protective power against *epidemic and contagious* disease. Among other examples in proof of this he cites the case of Howard the philanthropist, who passed unscathed through so many pestilential dangers. Doubtless Howard's temperance and moral courage greatly conduced to his long immunity, but we must not forget he at last fell a victim to his devotion to the cause of humanity. Then, again, the progress of the cholera in New

York is brought in evidence; because a Mr. Graham induced several persons there to adopt a vegetable diet, none of whom fell victims to the disease. Loose evidence of this kind is of no avail in these days of statistics and particulars; and we wish to know how the author explains, upon his vegetable feeding hypothesis, the fact that London, the most flesh-consuming of all the capitals attacked, suffered the least; and that that portion of its community which is most addicted to anti-Pythagorean practices was precisely the portion that escaped nearly scot-free. The low ill-fed population of Dublin perished in vast numbers, as have repeatedly done the vegetable-feeders of India.

Two chapters are devoted to proving that the employment of vegetable food is conducive to the symmetry and development of the body, and to the acuteness and perfection of the special senses; while for those who may believe that, however useful, it cannot be the most pleasant of food, Mr. Smith has also words of consolation.

"The prevalent notion that vegetable diet requires the continual exercise of self-denial, and considerably diminishes the pleasures arising from the gratification of the palate, is with many persons the most weighty objection to any change. This notion is however decidedly erroneous; and not one fact can be brought forward in support of it. Under a fruit and farinaceous diet, the organs of smell and taste become much more sensitive; and the sensations resulting from the gratification of a natural appetite upon this food, are much more exquisite and refined, and much more constant, than can possibly be experienced on an animal or mixed diet; which requires great variety, continual changes, and many additions from the vegetable world, to prevent disgust. I do not assert that the vegetable-eater looks forward with such anxious cravings to an expected meal, as the bon-vivant; who has, perhaps, been under the necessity of rousing the appetite of an already surfeited system, by bitters and stimulants; this would be to withdraw his attention from higher and more worthy pursuits; and to convert the supreme enjoyments of a moral and intellectual being, into the inferior pleasures of the sensualist. * * * * * * * * It is true that some who have but lately adopted a vegetable diet, meet with many tempting dishes of animal food, which they find it difficult to deny themselves; and to persevere requires a firm resolution, and a mind well convinced of the advantages to be derived from the change: but when time has familiarized the organs to more simple preparations, and the mind has been fully satisfied as to the propriety of the change, and the benefits resulting from it, the former dishes will become more offensive than pleasing, and the perfumes arising from them will only increase the disrelish for them. Since my commencement of vegetable regimen, I have several times partaken of animal food again, for the purpose of making observations on the change affected by diet in the secretions, &c.; and always had considerable difficulty in overcoming the disgust which the taste of flesh at first excited. The pulse was accelerated, the breathing became more rapid, the temper more irritable, the mind less collected, and the feelings generally less comfortable. In the course of two or three days the antipathy was overcome, and the other effects gradually subsided, but not entirely; and I always returned to a more simple and a more natural diet, with great pleasure, as upon it my enjoyment was much more complete." P. 327—9.

Numerous authorities are quoted to prove that vegetable diet is *favourable to mental exertion and intellectual culture;* while the effects of animal food in implanting bad passions and propensities the author holds as quite proven. Did not Fuseli and Mrs. Ratcliffe devour raw meat when they wished to engender an extra supply of the horrible in their imaginations? And few parents are aware of the mischief they are inflicting on the moral constitution of their children by allowing them, or rather forcing them, to partake of animal food in any shape.

*Vegetable Diet favourable to Longevity.*—Mr. Smith maintains that, with a vegetable regimen, not only is the enjoyment of life much increased, but its duration is also much more prolonged; for not only have most of the celebrated very long livers been entirely or chiefly vegetable feeders; but the fact of the more rapid renewal of tissue, which takes place when employing an animal diet, is significative of a more speedy wearing-out of the bodily machine. Temperance and exercise have always been the two great means for attaining longevity; and in what manner a moderate share of animal food could prove an impediment to this it would be difficult to imagine.

So much as regards the individual; but Mr. Smith's vegetable regimen has rendered him too much of a philanthropist and philosopher to allow his ruminations to stop at this point, and accordingly he carries his onslaught upon beef and mutton into the realms of political economy. From evidence derivable from this quarter he does not hesitate to predict that, at some future time, *nolens-volens*, vegetable food *must* become the universal food of man—albeit, that such day may be hundreds or thousands of years distant. When population has so increased, that space will have to be economized in order to produce the means of feeding it, then the author flatters himself men will be at last driven to feed on that which is after all best fitted for them. Liebig has calculated that if a man accustomed to subsist upon animal food and starch were to discontinue the latter, he would have to consume five times the quantity of the former—so that one pound of starch seems to supply the place of four pounds of flesh.

"If it be admitted, that an average of six pounds of animal food a day would be necessary for each individual, on an *exclusively flesh diet*, then, since an acre of land employed in feeding cattle only produces 10 ounces of flesh per day, it would require nearly 10 acres to support each person for a year; whereas one acre of wheat would supply three persons, and (according to Curwen) one acre of potatoes would serve at least 10 persons with sufficient food for the same space of time:—so that a diet of potatoes and fruit would support 100 times the number of inhabitants that could be maintained on an *exclusively flesh diet.*

"In the United Kingdom of Great Britain and Ireland there are at present about 28 million inhabitants, and about double the number of acres of land in cultivation; consequently two acres for each individual. Were all living on a full animal diet, the land could only supply food for five million six hundred thousand inhabitants; on the greatest delicacies of fruit, grain, and roots, 112 millions; on grain and other vegetables (when according to Lauderdale one acre will support four persons) 224 million; on potatoes and common fruits, 560 million:—without including the extra produce from improved culture. Or, let us suppose that, in Great Britain and Ireland, there are (in round numbers) 80 million acres; of which 60 million are arable, or capable of being cultivated. Let half of these be appropriated to the production of the finest fruits, flours, and timber, and to the support of cattle, sheep, and other animals for the production of milk, wool, &c.; we shall then have 30 million acres for potatoes, wheat, and other grain. Let one-half of this remnant be sown with wheat, and the remaining fifteen million planted with potatoes: then—

| | |
|---|---|
| 15,000,000 of acres of wheat, at 3 qrs. per acre, will feed | 45,000,000. |
| 15,000,000 of acres of potatoes, at 10 persons per acre . | 150,000,000. |
| | 195,000,000 |

which is equal to seven times the present population, and more than thirty times the number that the land would support upon flesh alone." P. 391-2.

As our population doubles itself every 50 years, lest any of our carnivorous readers may feel startled at the author's figures and predictions, and have fears for the good dieting of their descendants; let us hasten to express our conviction that so determined is the predilection of John Bull for beef and mutton, that have it he will; and if he cannot grow it upon his present large surplus of millions of unemployed acres, he will employ those facilities which a long purse, freedom of commerce, and improved means of transport, will furnish him with, for inducing other nations to devote some of their boundless territories to the satisfying his carnal cravings. Mr. Smith believes that our country has now reached or thereabouts its zenith, and if we are to prevent that period of decay which has hitherto crumbled down the proudest empires, this will be accomplished by the universal adoption of a natural diet—when the richer classes will consent to forego their present privileged condition—not as regards political or social rights, but in respect to dietetic rights, which at present allow "the rich man to consume the produce of an acre at a meal, while his unfortunate brother is left to starve."

The work is terminated by a few rules for those who are about to commence an exclusively vegetable diet. In the first place they must be determined and persevering, and should conduct the change gradually, allowing a few weeks to elapse in the process of accustoming themselves. Due mastication and insalivation are more important here than when feeding on a mixed diet. Each meal must be completely digested before another is taken. If a desire for food recurs sooner than six hours, it is an indication of imperfect chylification. The author enumerates the various articles whence the diet may be selected; but these we need not particularize, as all the grains, roots, and fruits are among them. He seems to attach more importance to the last as articles of diet, than any other writer we have met with. He repeatedly, in the course of his work, refers to the error of those who consider herbs and greens, and such-like aliments, as forming important articles of the vegetable-eater's diet. Nevertheless we should much like to have seen Mr. Smith's own daily bill of fare; for in spite of all his encomia, we cannot but think the farinaceous and fructaceous classes of aliments form but a monotonous diet after all.

In conclusion, we may express the opinion that Mr. Smith has stated his case ably but not successfully: he has said enough in favour of an exclusively vegetable diet to shew that it offers all the requisites for human sustenance when fancy, ill-health, or any particular circumstance, may induce its adoption; but no sufficient arguments or experience have been advanced to lead us to believe that any advantage would arise from its substitution in place of the ordinary mixed regimen. His statements rather apply to an exclusively flesh-diet, which no one supports, and than which the vegetable diet would be infinitely preferable; and if any of them are of at all an extensive applicability, they are so only to those classes who habitually indulge in too large a proportion of animal food, which is certainly the case with many of the wealthy and inactive inhabitants of cities. Mr. Smith, as we have seen, believes animal food is even a great provoker of disease, and cites with pleasure the fact of so large a portion of the human race being able to dispense with it. So far from agreeing with him in this view, we confess to the opinion that much of the Englishman's comparative good health and robust constitution, much of his laborious application, indomitable courage, and unwearied perseverance, arise from the superior *stamen* his more substantial diet confers upon him. He is the greatest meat-eater among the livers upon a mixed diet, and yet who will venture to assign him an inferior position in the scale of nations. We believe his retention of his present rank in that will be much dependent upon his continuing to obtain a sufficient, but not an excessive, supply of the "Roast Beef of Old England."

We do not think Mr. Smith will make many proselytes, but to all those whose condition of health, temperament, or stings of conscience, may render the resorting to a vegetable diet desirable, we can recommend his book as saying all that can be said in its favour.

